# Gender Differences in Surgical Case Volume Among Neurosurgery Residents

**DOI:** 10.7759/cureus.35798

**Published:** 2023-03-05

**Authors:** Kyle M Rei, Vedhika Reddy, Sumayya Mohammed, Samir Kashyap, Alessandra Cathel, Javed Siddiqi

**Affiliations:** 1 Neurological Surgery, California University of Science and Medicine, Colton, USA; 2 Neurology, Arrowhead Regional Medical Center, Colton, USA; 3 Neurosurgery, Arrowhead Regional Medical Center, Colton, USA; 4 Neurosurgery, Desert Regional Medical Center, Palm Springs, USA; 5 Neurosurgery, Riverside University Health System Medical Center, Moreno Valley, USA; 6 Neurosurgery, California University of Science and Medicine, Colton, USA

**Keywords:** surgical case volume, residency, neurosurgery, gender disparity, gender differences

## Abstract

Objectives: Gender differences in surgical training opportunities, measured by case volume, have been demonstrated in the fields of otolaryngology and ophthalmology. We hypothesize that this gender disparity is not present among neurosurgery residents. This study compares median female and male case volumes stratified by postgraduate year (PGY) level for U.S. neurosurgery residents.

Methods: This retrospective analysis included case log data from two southern California neurosurgery residency training programs, Riverside University Health System (RUHS) and Desert Regional Medical Center (DRMC), from 2015 to 2021. For each PGY level, gender differences in case volumes were summarized using median, SD, and two-sided t-tests.

Results: Among 47 (19.1% female) neurosurgery residents, there were no significant gender differences in case volumes across any PGY levels. Female residents had greater median surgical cases during PGY-1 (median (SD), female 107.0 (13.1) vs male 102.0 (24.3); p=0.841) and PGY-7 (female 282.5 (17.7) vs male 246 (60.9); P=0.424), while male residents had greater median case volumes for all other PGY levels.

Conclusions: Although previous studies have found significant gender differences in case volumes among surgical residents in otolaryngology and ophthalmology, case log data from two neurosurgery residency programs in southern California, RUHS and DRMC, does not reflect this gender disparity at any PGY level.

## Introduction

Active male physicians (63.7%) outnumber female physicians, and this gender disparity is further magnified in surgical specialties [1]. In 2019, 22% and 9.3% of general surgeons and neurosurgeons were female, respectively [1]. However, there is evidence of a changing tide with 2020 marking the first year that more women graduated from U.S. MD-granting medical schools than men. Furthermore, in 2021, 20.5% of neurosurgery residents were female, which is more than twice the gender representation of active female neurosurgeons [2,3].

Beyond demographic disparities, there is also a debate about whether female residents in surgical specialties receive an equal quality of training compared to their male counterparts [4-6]. A common method of measuring differences in training opportunities is by comparing surgical case volumes; if one gender group has significantly lower average case volumes, this could indicate an institutional bias [4-6]. Existing literature has explored these differences in the fields of otolaryngology and ophthalmology, however, to our knowledge, there have not been any studies investigating training parity in neurosurgery residency programs.

In this study, we aim to examine gender differences in training opportunities, measured by resident case volumes, at two neurosurgery residency programs in southern California: Riverside University Health System (RUHS) and Desert Regional Medical Center (DRMC).

## Materials and methods

This retrospective study analyzed 47 (19.1% female) de-identified case logs pooled from all the residents enrolled in neurosurgery programs at RUHS and DRMC from 2015 to 2021. Case log data included resident gender, postgraduate year (PGY), academic year, rotation location, non-surgical rotations, and total surgical case volume per PGY level. Residents self-reported their case volumes by logging completed procedures into the Accreditation Council for Graduate Medical Education (ACGME) Case Log System. Consequently, four outliers emerged from the data with fewer than 50 completed cases in a given PGY level: three male PGY-1 logs and one male PGY-2 log. These outliers were removed from the data and were not found to impact the significance. Microsoft Excel (Microsoft Corporation, Redmond, WA) was used to calculate descriptive statistics and perform two-sided student’s t-tests with a significance level of <0.05. The primary study outcome was differences in median surgical volume between female and male residents of equal PGY levels. The Institutional Review Board in connection with Arrowhead Regional Medical Center approved this study and did not require informed consent for the use of deidentified data.

## Results

**Figure 1:**
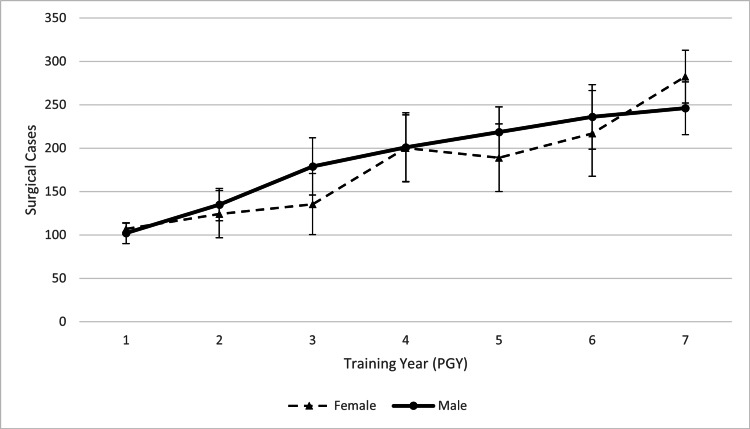
Median surgical cases by PGY level PGY, postgraduate year

Case logs from 47 (19.1% female) neurosurgery residents trained at DRMC and RUHS from 2015 to 2021 included 31,793 surgical cases (Table 1). DRMC case logs included 40% female residents, while RUHS case logs included 17.9% female residents. For residents currently enrolled in these programs, data was included up to the highest fully completed PGY level of training. Data from a single resident is reflected in each PGY level of training he or she completed at their respective program (Table 2). Total case volume increased each year of training, on average (mean (SD), PGY-1 103.7 (22.4) vs PGY-7 263.9 (51.8); P<0.001) (Figure 1). Therefore, case volumes were stratified by PGY level when comparing female and male residents to isolate gender differences in training from gender differences in representation at each PGY level (Table 3). This study found no significant gender differences in case volumes across any PGY level. Female residents had greater median surgical cases during PGY-1 (median (SD), female 107.0 (13.1) vs male 102.0 (24.3); p=0.841) and PGY-7 (female 282.5 (17.7) vs male 246.0 (60.9); p=0.424), while male residents had greater median case volumes for all other PGY levels. An average effect size (Cohen’s d) of 0.251 was found across all PGY levels.

**Table 1:** Sample size (n) by training institution and gender DRMC, Desert Regional Medical Center; RUHS, Riverside University Health System

	Female Residents (n)	Male Residents (n)
Total	9	38
DRMC	4	10
RUHS	5	28

**Table 2:** Sample size (n) by training year and gender PGY, postgraduate year

Training Year	Female	Male
PGY-1	5	21
PGY-2	6	25
PGY-3	6	26
PGY-4	7	22
PGY-5	4	20
PGY-6	4	19
PGY-7	2	5

**Table 3:** Comparison of median surgical cases (SD) by training year PGY, postgraduate year

Training Year	Female (SD)	Male (SD)	P Value
PGY-1	107 (13.1)	102 (24.3)	0.841
PGY-2	124 (54.6)	135 (37.0)	0.622
PGY-3	135.5 (70.2)	179 (65.8)	0.897
PGY-4	200 (76.5)	201 (79.4)	0.547
PGY-5	189 (77.7)	218.5 (58.1)	0.587
PGY-6	217 (99.1)	236 (74.1)	0.821
PGY-7	282.5 (17.7)	246 (60.9)	0.424

## Discussion

This study examined 47 case logs from neurosurgery residents from 2015 to 2021 and found no significant gender differences in case volumes at any PGY level. Female residents had greater median surgical case volumes at the PGY-1 and PGY-7 levels compared to their male counterparts. These findings are in contrast with previous work in the fields of otolaryngology and ophthalmology (Table 4).

**Table 4:** Studies investigating case volume gender variance *Positive value indicates male > female

Author	Year	Specialty	Case Volume Gender Variance*	p
Gurgel	2020	Otolaryngology	34.8	<0.001
Gong	2019	Ophthalmology	58.1	<0.001
Gill	2021	Ophthalmology	178	0.036

Beginning with training parity among otolaryngology residents, Gurgel et al. reviewed 2,544 resident case logs (32% female) from 2009 to 2017 and found that across all years, female residents performed 34.8 fewer cases per graduating year than their male peers on average (95% CI, 19.4, 50.2; p<0.001) [4]. However, this finding should be cautiously interpreted with the context that this disparity does not suggest a gap in competence, as residents must perform a minimum number of cases to graduate as determined by the ACGME [7].

Similarly, in the field of ophthalmology, Gong et al. analyzed 1,271 resident case logs (36% female) from 24 programs, from 2005 to 2017, and found that female residents on average performed 58.1 fewer total cases than their male peers (95% CI, 36.0 to 80.2; p<0.001) [5]. This study also considered the potential confounder of parental leave. Since trainees taking leave may perform fewer total cases, the disparity could be attributed to one gender group having a higher proportion of trainees who elect to take leave. This study found that the proportion of residents who took parental leave was similar between gender groups: 10.4% male and 15.6% female. This subgroup of male residents performed an average of 27.5 more cataract operations than the male residents who did not take paternity leave (95% CI, 13.3 to 41.6; p<0.001). Among female residents, those who took maternity leave performed on average 2 fewer operations than those who did not take maternity leave (95% CI, −18.0 to 14.0; p<0.001). These results indicate that parental leave does not adequately account for gender differences in surgical training experiences.

Looking beyond the U.S., Gill et al. examined 241 ophthalmology trainee logbooks (40.7% female) from 2008 to 2020 from the Royal Australian and New Zealand College of Ophthalmologists and found that female trainees performed on average 178 fewer surgeries than their male peers throughout four years of training (p=0.036) [6]. Similarly, this study investigated the potential confounder of parental leave, and although 30.6% of females had interrupted training compared to 0.7% of males, parental leave or duration was not found to be significant (p=0.206).

Surgical case volume is of particular interest because in evaluating training parity among residents, it provides an objective, quantitative measure. The goal of surgical training is to transition residents from a position of low to full autonomy, and participating in more cases contributes to developing the confidence required to make such a transition [4]. It has been shown that larger case volumes are positively correlated with resident confidence and perception of receiving adequate training [8]. However, a lack of training parity should not be interpreted as posing a potential harm to patients because the training still must meet the ACGME requirements to demonstrate competence [7].

A strength of this paper is that the data collectively represents 10 unique institutions. Residents enrolled in the DRMC program rotate at seven sites: (1) DRMC, (2) Arrowhead Regional Medical Center, (3) Redlands Community Hospital, (4) Children’s Hospital Los Angeles, (5) Kaiser Anaheim, (6) San Diego Naval Medical Center, and (7) Kaiser Fontana. Similarly, residents enrolled in the RUHS program rotate at six sites: (1) RUHS, (2) Arrowhead Regional Medical Center, (3) Kaiser Fontana, (4) Kaiser LA, (5) Children’s Hospital Los Angeles, and (6) Loma Linda University Medical Center.

If a gender disparity had been found in case volumes, a common hypothesis is that institutional biases negatively impact the training female residents receive [4-6]. Because this data represents case logs from residents at 10 unique training institutions across Southern California, our results suggest a lack of uniform and pervasive institutional bias across the entire region. However, if one of these training institutions did, in fact, suffer from institutional bias, then it is unlikely that it would be captured in these findings.

A limitation of this paper is the sample size. The authors requested deidentified national data from the ACGME, and the request was not granted. Consequently, these findings have limited generalizability, and we encourage other neurosurgery programs to publish their case log statistics. Another limitation is that case data is self-reported, as is true for all residency programs, which incorporates potential error as residents may neglect to report every case they participate in. In addition to case volume, considering the resident’s role in each case would have added further insight into the true training parity; however, this level of detail was not made available. For purposes of comparison, case volume was a useful analysis to add to the growing body of literature. Finally, previous studies investigated whether parental leave was a confounding variable that could explain case volume differences between the genders, and this was not a focus of our investigation. However, across multiple studies, parental leave was not found to be significant [5,6].

Although we found no significant disparities in our investigation of case volumes, we recognize that gender disparities may manifest in other ways within the field of neurosurgery. Notably, beyond demographics, case volumes, and other quantitative metrics, there are qualitative experiences that one may only capture in the survey of perceptions. For instance, in a 2020 survey of 870 neurosurgeons (23.6% female), it was found that female respondents were less likely to feel acknowledged for their ideas (p<0.001) [9]. Female respondents also reported that they were less likely to feel that they received equal treatment from supervisors compared to their male counterparts (p<0.001). Additionally, 83.3% of female respondents believed their gender was an obstacle in career advancement, compared to 10.3% of male respondents (p<0.001) [9].

These perceptions of gender disparity in neurosurgery could be attributed to a heavily male-dominated organizational culture, which informs attitudes and beliefs about recognition and growth within the field [10]. It has been found that within professional settings, women are more likely to believe that they are expected to be tolerant and reflective, while men are more likely to believe that they are expected to be aggressive [11]. Furthermore, women, at a population level, tend to be higher in trait agreeableness, while men tend to be higher in trait aggressiveness [12]. In the context of surgical training, these findings could, in part, explain the lack of training parity found in previous studies if it is also found that male residents have a greater tendency to participate in cases beyond those directly assigned. Further research could evaluate this hypothesis. Since our data includes 23.7% female residents, which is more than twice the representation of active female neurosurgeons, this could reflect a less extreme male-dominated organizational culture that fosters greater parity.

Based on the findings of this study, we encourage other neurosurgery programs to investigate their case logs to ensure training parity among its trainees. Additionally, surveying perceived training equality could serve to help programs develop inclusive organizational cultures despite demographic asymmetries. Areas for further research include (1) investigating whether gender differences in trait assertiveness manifest in surgical training programs with male residents having a greater tendency to participate in extra, unassigned cases than their female counterparts, and (2) whether personality differences across specialties could account for the inconsistent results found by this neurosurgery study compared to previous studies in otolaryngology and ophthalmology.

## Conclusions

Although previous studies have found significant gender differences in case volumes among surgical residents in otolaryngology and ophthalmology, indicating a lack of gender parity in training opportunities, case log data from two neurosurgery residency programs in southern California, RUHS and DRMC, does not reflect this gender disparity at any PGY level. Due to data limitations, this finding should serve as an invitation for further replication and validation.

## References

[1] (2022). Association of American Medical Colleges: table 1.3 number and percentage of active physicians by sex and specialty, 2019. Specialty.

[2] (2022). Association of American Medical Colleges: table B-2.2: total graduates by U.S. MD-granting medical school and sex, 2016-2017 through 2020-2021. https://www.aamc.org/media/6111/download.

[3] American Association of Medical Colleges: Table B3 (2022). American Association of Medical Colleges: table B3. number of active residents, by type of medical school, GME specialty, and sex 2020-21 active residents. https://www.aamc.org/data-reports/students-residents/interactive-data/report-residents/2021/table-b3-number-active-residents-type-medical-school-gme-specialty-and-sex.

[4] Gurgel RK, Cardon BR, Allen CM (2020). Evaluating gender parity in operative experience for otolaryngology residencies in the United States. Laryngoscope.

[5] Gong D, Winn BJ, Beal CJ (2019). Gender differences in case volume among ophthalmology residents. JAMA Ophthalmol.

[6] Gill HK, Niederer RL, Danesh-Meyer HV (2021). Gender differences in surgical case volume among ophthalmology trainees. Clin Exp Ophthalmol.

[7] Baugh TP, Franzese CB (2017). Extremes in otolaryngology resident surgical case numbers: an update. Otolaryngol Head Neck Surg.

[8] Suwanabol PA, McDonald R, Foley E, Weber SM (2009). Is surgical resident comfort level associated with experience?. J Surg Res.

[9] Gadjradj PS, Matawlie RH, Voigt I, Harhangi BS, Vleggeert-Lankamp CL (2020). Gender differences between male and female neurosurgeons: is there equality for all?. World Neurosurg.

[10] Lemkau JP (1983). Women in male-dominated professions: distinguishing personality and background characteristics. Psychol Women Q.

[11] Bellou V (2010). Organizational culture as a predictor of job satisfaction: the role of gender and age. Career Dev. Int..

[12] Costa PT, Terracciano A, McCrae RR (2001). Gender differences in personality traits across cultures: robust and surprising findings. J Pers Soc Psychol.

